# A technique for laparoscopic extraperitoneal colostomy with an intact posterior sheath of rectus

**DOI:** 10.1186/s12893-022-01686-w

**Published:** 2022-06-20

**Authors:** Zeyu Li, Lifei Tian, Ruiting Liu, Bobo Zheng, Ben Wang, Xu Zhao, Pan Quan, Jian Qiu

**Affiliations:** Department of General Surgery, Shaanxi Provincial People’s Hospital, Xi’an, 710068 Shaanxi People’s Republic of China

**Keywords:** Extraperitoneal colostomy, Parastomal hernia, Colostomy, Rectal cancer

## Abstract

Regardless of the advances in surgical techniques, parastomal hernia is still an inevitable complication for many patients with low rectal cancer undergoing abdominal perineal resection (APR). Extraperitoneal colostomy (EPC) seems to be a effective method to reduce the risk of parastomal hernia. We propose a new approach to simplify and standardize laparoscopic EPC to make this operation easy to perform. We used the technique of laparoscopic TEP groin hernia repair to produce an extraperitoneal tunnel, which can not only facilitate precise visualization of the extraperitoneal tunnel but also utilize the intact posterior rectus abdominis sheath as biologic materials to maintain soft-tissue augmentation, with a satisfactory result. With laparoscopy, we can create adequate space without insufficient dissection of the extraperitoneal tunnel while avoiding damage to the retrorectus sheath. At the time of writing, we had performed this method in four patients, without any complications. This technique is effective at preventing parastomal hernia without extra costs.

## Introduction

Regardless of the myriad of available sphincter-preserving techniques, abdominal perineal resection (APR) is still a radical and preferred treatment option for approximately 10–20% of patients with low rectal cancer [1]. Although studies on tumor resection have been widely reported, those on colostomy formation techniques have received considerably less attention. In the traditional method, a permanent stoma is created after APR through transperitoneal colostomy (TPC), but this approach is accompanied by a very high risk of parastomal hernia. To reduce parastomal hernia, surgeons have invented extraperitoneal colostomy (EPC), which is reported to have a lower rate of parastomal hernia than the transperitoneal route [2]. However, laparoscopic EPC remains a challenge for surgeons and is regarded as a time-consuming and highly technical process.To address these issues, we therefore propose a new approach to simplify and standardize laparoscopic EPC to make this operation easy to perform.

## Methods

### Patients and study design

Since January 2021, 16 patients with rectal cancer underwent laparoscopic APR at Shaanxi Provincial People’s Hospital in China. We analyzed retrospectively the records of 16 of these patients, who were followed up for over 6 months. Four of the 16 patients were operated on using our new technique. This study included the patients’ demographics (age, sex, body mass index, comorbidity, stage of tumor, History of abdominal surgery, Surgical/postoperative outcomes (operation time, Colostomy time, surgical complications). Thereafter, patients were periodically assessed during follow-up outpatient visits or with radiological examinations.

### Patient preparation

Before the operation, our stoma therapist marked the site of colostomy, which was located just across the left rectus abdominis muscle at or slightly below the level of the umbilicus. Standard operative protocols were utilized. The patients were given general anesthesia in the lithotomy position. Surgical-related anatomy, such as the xiphoid process, bilateral subcostal margins, linea alba, rectus muscle, and semilunar lines, was marked.

### Port position for the extraperitoneal tunnel with laparoscopic totally extraperitoneal (TEP) technique

**Fig. 1:**
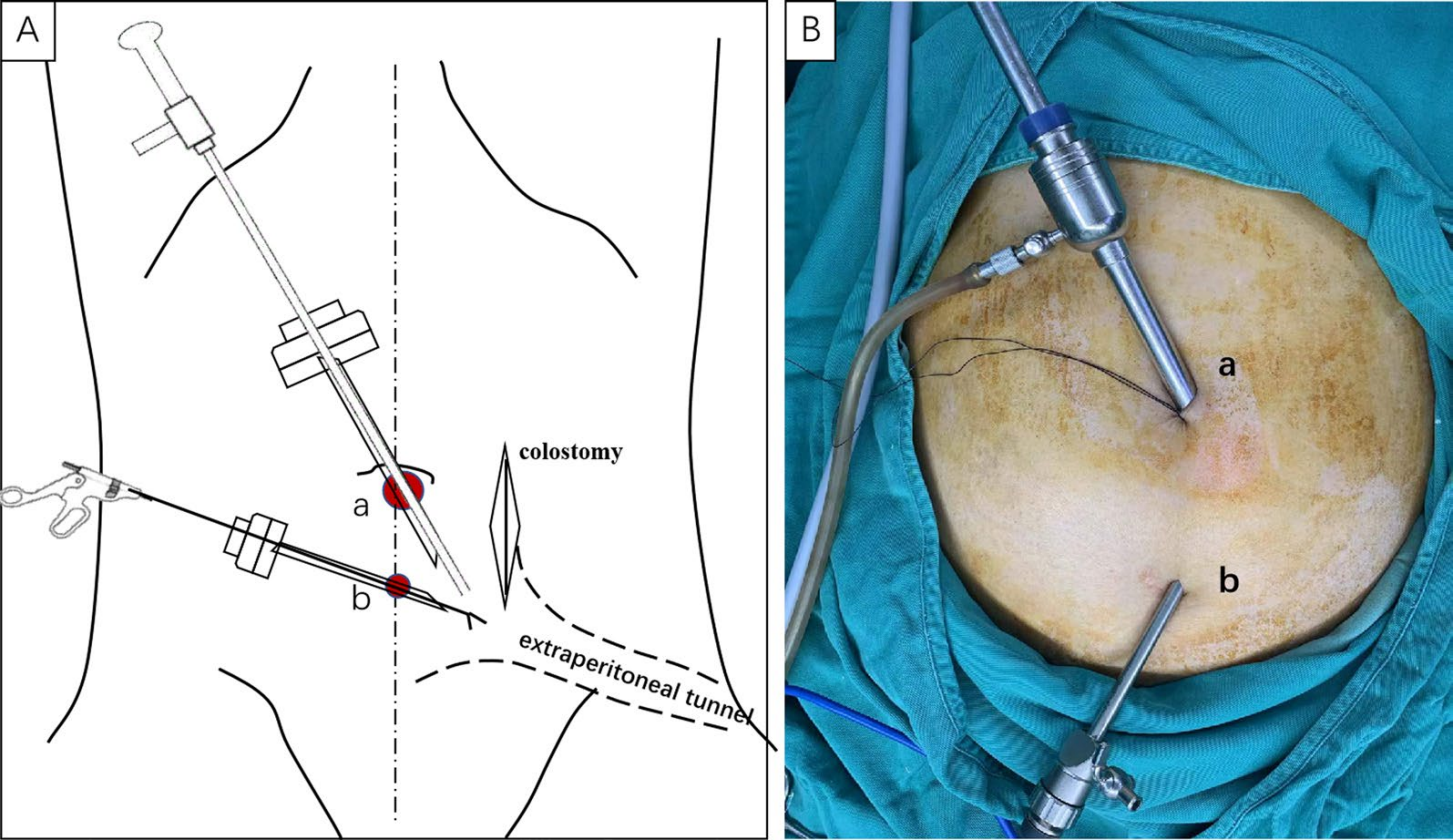
**A** Surgical technique of stoma creation through the extraperitoneal route and **B** trocar placement

**Fig. 2:**
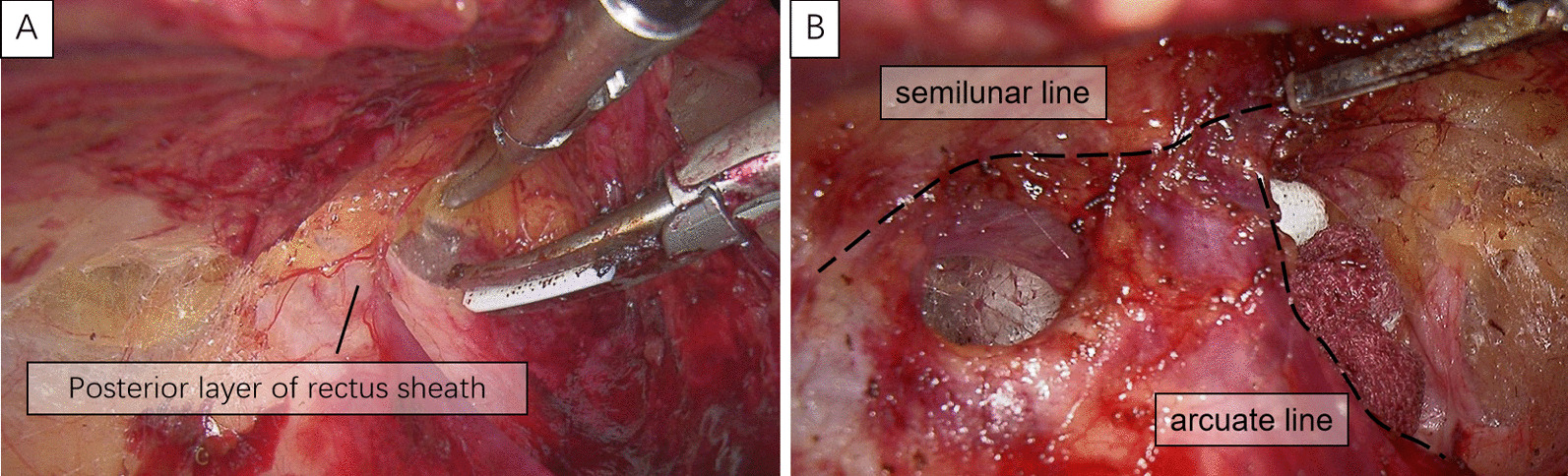
Dissection of the extraperitoneal tunnel under visualization (**A**). Separation of the space between the rectus abdominis and posterior rectus sheath (**B**)

**Fig. 3:**
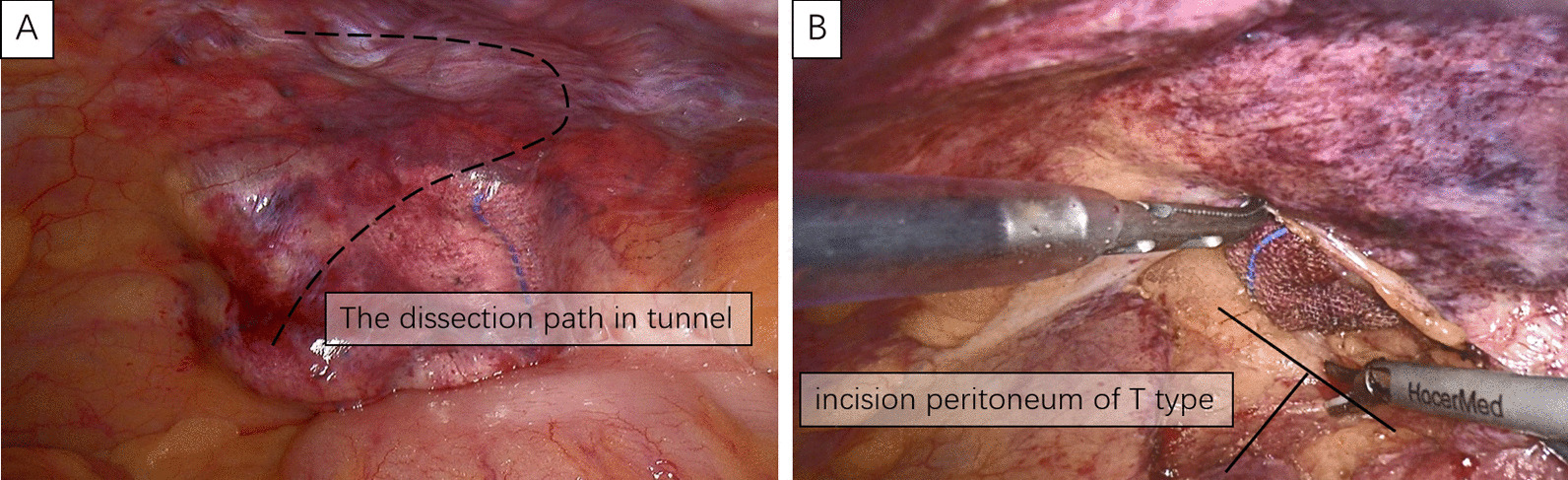
The extraperitoneal tunnel was separated into an inverted “L” type (**A**), and the abdominal cavity was opened into a “T” type (**B**)

A 1-cm transverse incision was made in the midline of the abdomen, just below the umbilicus. After blunt dissection of the anterior rectus fascia, we opened the layer between the rectus muscle anteriorly and the posterior rectus fascia posteriorly with a small blunt hook. Then, a 10-mm trocar (port A) was slowly inserted into this plane and advanced along the midline to the pubis under direct visualization. Blunt dissection was performed using the laparoscope with pneumoperitoneum pressure maintained at 12 mmHg to obtain enough space to insert the other trocars. A 5-mm trocars were subsequently inserted in the midline of the abdomen 5-cm (port B) below port A, providing a distance of one another to minimize mechanical interference (Fig. 1). The space was expanded outward and downward to the lateral edge of the posterior sheath without damaging the sheath to create an inverted L-shaped tunnel. In addition, the rectus sheath, at 3 cm below the lower edge of the colostomy, was kept intact to ensure adequate support for the stoma colon. Then, a 5-cm incision was made at the lateral edge of the posterior sheath into the extraperitoneal space (Fig. 2). The extraperitoneal space was separated and expanded using a harmonic scalpel between the transversalis fascias and the transverse muscles, thereby creating a 5-cm-diameter extraperitoneal tunnel. Aseptic gauze was placed at the end of the intraperitoneal tunnel to confirm the dissecting position of the peritoneum (Fig. 3). It should be noted that the extraperitoneal tunnel must be established before abdominal perineal surgery to avoid intraoperative tumor dissemination.

### Extraperitoneal colostomy

**Fig. 4:**
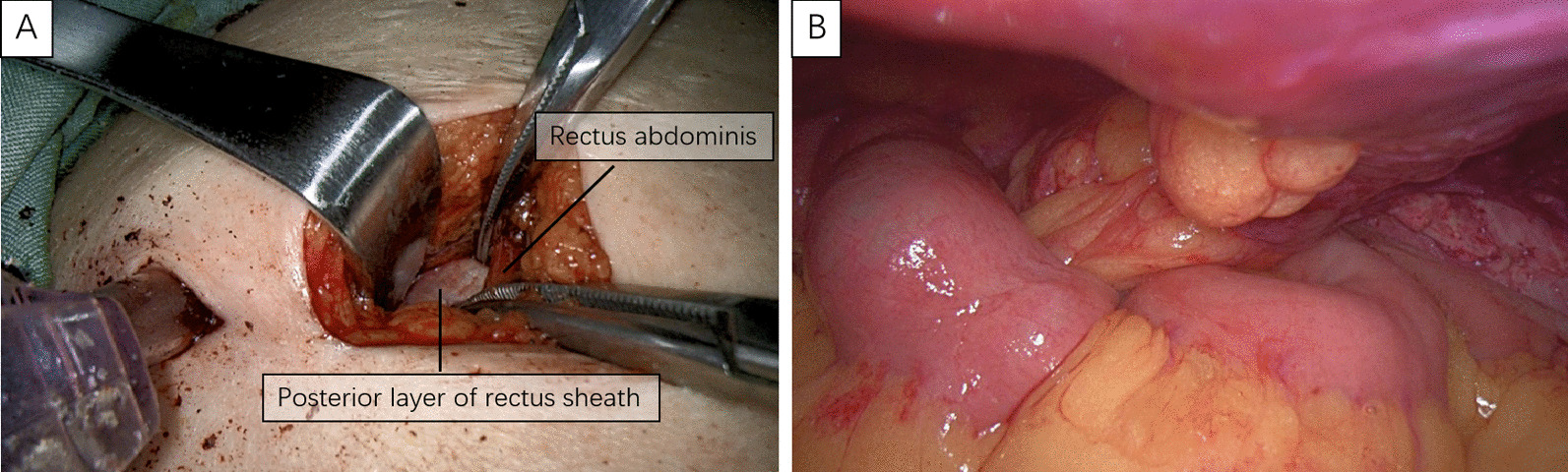
The skin was cut at approximately 3 cm in diameter **A** and the proximal sigmoid colon was pulled out of the tunnel (**B**)

APR was performed via the standard operation. The remaining length of the sigmoid colon was moderate to avoid intestinal prolapse. After APR completion, a 5-cm incision was made in the peritoneum to allow the colon to pass smoothly (Fig. 4). At the premarked stoma site, the skin was cut at approximately 3 cm in diameter, and a cruciate incision was performed in the anterior rectal sheath. Finally, the proximal sigmoid colon was pulled out of the tunnel, and the intestinal wall and skin were sutured using the standard technique.

### Statistical analysis

The measurement data were shown as median [Q1, Q3], and categorical data were shown as the number (percentage) in the group. Statistical analysis was conducted using SPSS (version 19). A P value of < 0.05 was considered to indicate statistical significance. Continuous variables were assessed using Kruskal-Weiss test. Categorical variables are summarized as percentages, and were analyzed by the Chisquare test or Fisher’s exact probability test.

## Results

**Fig. 5:**
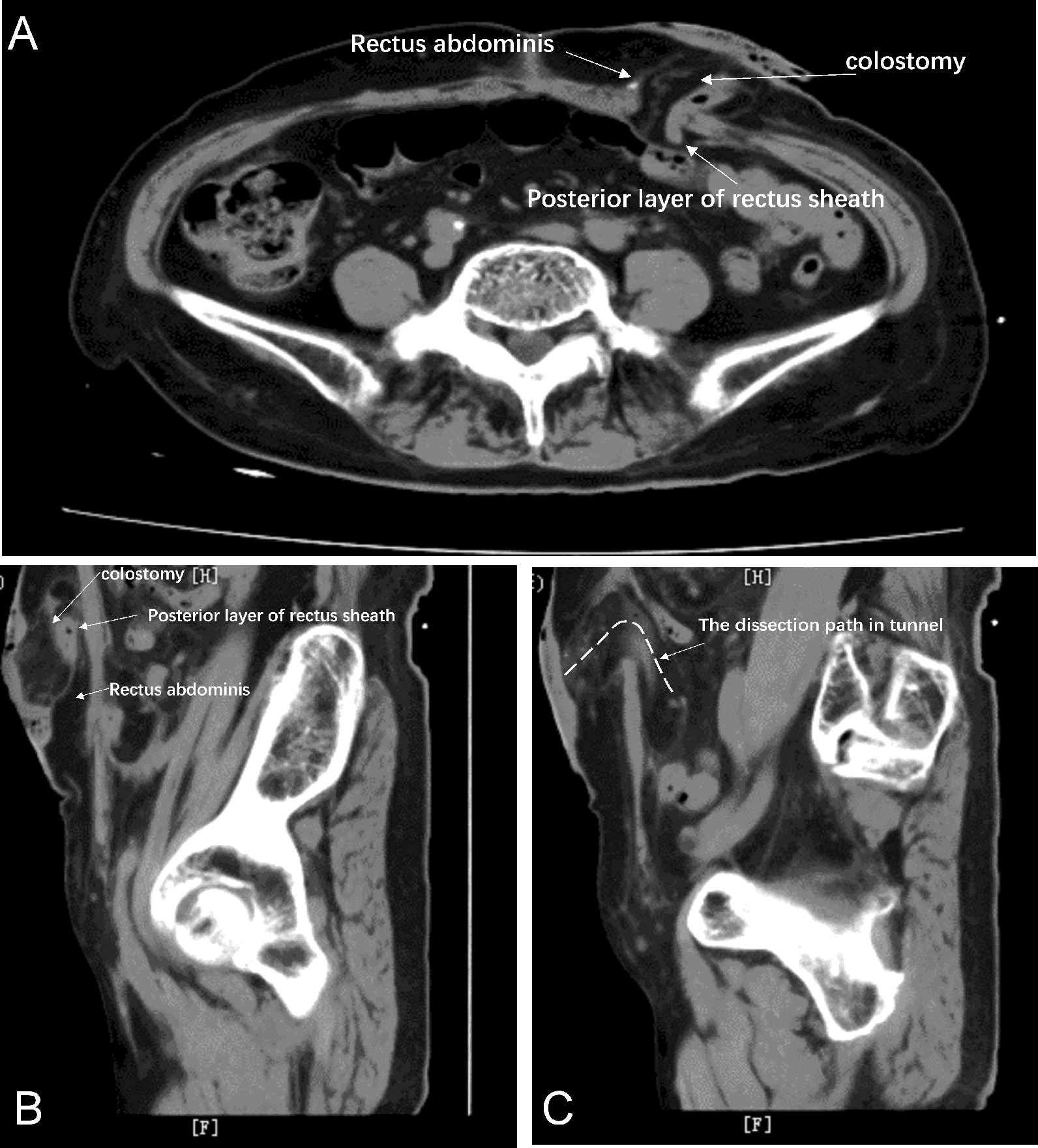
CT scan at 1 month after APR

All surgeries were performed by a single experienced colorectal surgeon. Since January 2021, our group has applied this technique for 4 cases and other 12 cases underwent the conventional EPC. There was no significant difference in Sex, Age and BMI between the EPC group and new technique group (Table 1). There was no significant difference in total operation time between the EPC group and new technique group. However, the time to create an extraperitoneal stoma tunnel using this new technique tend to be less than the time required for the conventional EPC (*P* < 0.05). There were no complications during the operation (Table 2). The diagnosis of parastomal hernia was confirmed by both physical and/or radiological examination. Clinical diagnosis was performed with the patient in the standing position; computed tomography (CT) was performed in the supine position while performing the Valsalva maneuver. Four patients received at least one CT scan at 30 days after the surgery. There was no parastomal hernia, either clinical or radiological, during at least 6 month postoperative follow-up period (Fig. 5).

## Discussion

As one of the most common complications after APR, parastomal hernias dramatically affect patient quality of life, ranging from parastomal discomfort or pain to life-threatening complications, such as intestinal strangulation, perforation and obstruction. An incidence of parastomal hernia (excluding stoma prolapse) up to 58% has been reported by systematic reviews with a maximum follow-up of 7 years [3, 4].Some studies have also revealed that the prevalence of a parastomal hernia of an end-sigmoid colostomy has been variously reported from 10–50% [5, 6]. Patients often demand complex reconstructive surgery, which performs a high recurrence rate. Many studies have reported the surgical techniques for parastomal hernia, such as the local use of various types of mesh [6, 7]. It does bring some good results to some extent, but creates some mesh-related morbidity as well. Because success in parastomal hernia repair has been limited, surgeons have focused on improving the method of end-sigmoid colostomy to reduce the occurence of parastomal hernia. Prevention is more important than treatment.

Recent studies have shown that preventive mesh placement and extraperitoneal stoma placement are both effective techniques to prevent parastomal hernia [8, 9]. The European Hernia Society (EHS) strongly recommends the utilization of prophylactic meshes to prevent parastomal hernias after APR. Some studies suggest that prophylaxis with synthetic mesh reinforcement of the stoma may reduce the risk of developing a parastomal hernia [10]. Manuel et al. [11] used a modified Sugarbaker technique with composite mesh to prevent parastomal hernia during laparoscopic abdominoperineal resection. However, this strategy still cannot avoid the risk of infection as well as short- and long-term risk of synthetic mesh erosion into the bowel or skin. James et al. [12] suggested the use of biologic materials to maintain the benefit of soft-tissue augmentation without the potential adverse consequences of synthetic materials. Brandsma et al. [13] further performed a multicenter randomized controlled trial using biological mesh to prevent parastomal hernia and obtained satisfactory results. However, it is not applicable to all patients because of the higher cost of treatment.

To reduce parastomal hernia, surgeons have invented extraperitoneal colostomy (EPC), which is reported to have a lower rate of parastomal hernia than the transperitoneal route [2]. A long-term study of permanent end-sigmoid colostomies reported a significantly lower risk of herniation with the extraperitoneal approach than the transperitoneal route (3.5% vs. 35%) [14]. Then, laparoscopic EPC was further devised and adopted from the corresponding technique of open APR [15, 16]. Several study groups have also shown that laparoscopic EPC substantially prevents the stoma-related complications seen in open EPC [9, 17, 18]. Nevertheless, these studies all had a nonrandomized design, indicating that more randomized controlled trials (RCTs) need to be performed to confirm whether the extraperitoneal route prevents parastomal hernia. Whether the peritoneum can provide adequate support also needs further study. In addition, laparoscopic EPC remains a challenge for surgeons and is regarded as a time-consuming and highly technical process. Zhang et al. [19] modified the EPC technique by keeping the posterior rectus sheath intact during colostomy, but ensuring visualization and precision and establishing a standard operational procedure for surgeons during the establishment of an extraperitoneal tunnel are difficult with this surgical method.

**Fig. 6:**
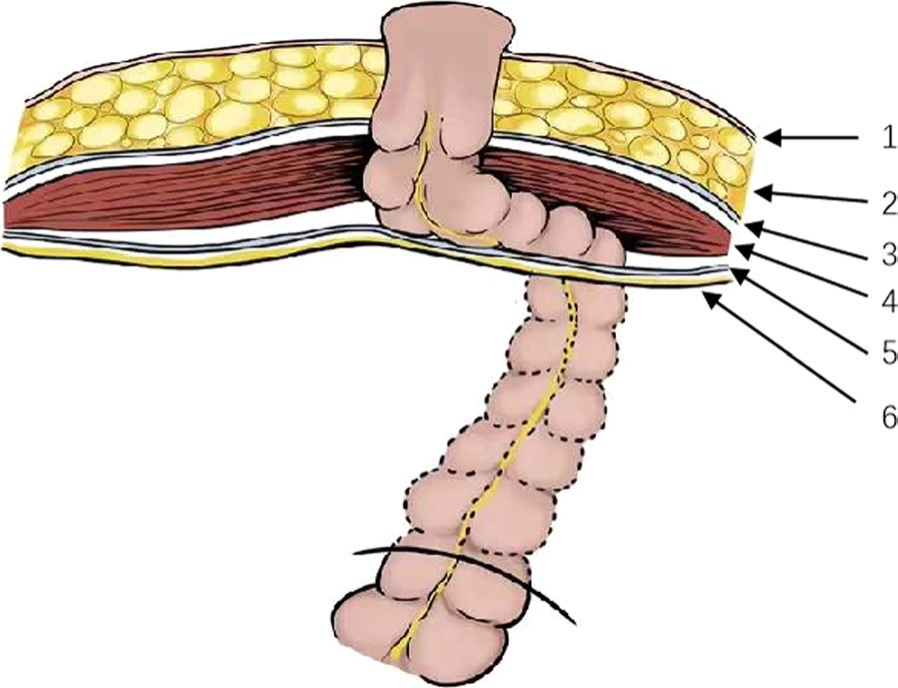
Longitudinal section diagram of the abdominal wall structure during extraperitoneal colostomy. 1-skin, 2-subcutaneous fat, 3-anterior rectus abdominis sheath, 4-rectus abdominis muscle, 5-posterior rectus abdominis sheath, 6-peritoneum

We used the technique of laparoscopic TEP groin hernia repair to produce an extraperitoneal tunnel, which can not only facilitate precise visualization of the extraperitoneal tunnel but also utilize the intact posterior rectus abdominis sheath as biologic materials to maintain soft-tissue augmentation, with a satisfactory result. With laparoscopy, we can create adequate space without insufficient dissection of the extraperitoneal tunnel while avoiding damage to the retrorectus sheath (Fig. 6). This new approach might be particularly useful for operating on patients with obesity or those with a history of abdominal surgery. But more data are needed for further study. It also does not increase operation time and costs. In our study, the follow-up times of the four patients were minimum 6 months, and all received CT scans at 1 month after APR. At the time of writing, no parastomal hernia had occurred in our patients. However, this technique requires further elaborate research and verification with more cases over a longer follow-up period.

## Conclusions

We describe a modified technique for extraperitoneal colostomy in laparoscopic operations, which maintains an intact posterior rectal sheath for soft-tissue augmentation. This technique is effective at preventing parastomal hernia without extra costs. These findings may contribute to the prevention of parastomal hernias.

**Table 1:** Patient demographics

Demographics	EPC (n = 12)	New technique group (n = 4)	*P* value
Sex (male/female)	4/8	0/4	NA
Age (years)	75.5 [67, 81.7]	70.2 [68.5, 72.6]	0.448
BMI (kg/m2)	23.8 [21.9, 26.1]	24.6 [20.9, 27.8]	0.673
Comorbidity, n (%)			
Diabetes mellitus	8 (66)	3 (75)	0.756
Hypertension	4 (33)	2 (50)	0.604
Stage of tumor, n (%)			
Stage II	1 (8)	1 (25)	
Stage III	6 (50)	2 (50)	
Stage IV	5 (42)	1 (25)	
History of abdominal surgery, n (%)	2 (16)	1 (25)	0.715

**Table 2:** Surgical/postoperative outcomes

	EPC (n = 12)	New technique group (n = 4)	*P* value
Operation time (min)	387.8 [327.7, 412.5]	366.9 [313.8, 397.4]	0.089
Colostomy time (min)	16.4 [15.3, 18.1]	13.7 [12.2, 15.8]	0.048
Complications, n (%)			
Parastomal hernia	0	0	NA
Stomal stenosis	0	0	NA

## Data Availability

The datasets of the current study are available from the corresponding author upon reasonable request.
